# Innate immune sensing and vaccine strategies against West Nile virus: role of Toll-like receptors and viral evasion mechanisms

**DOI:** 10.3389/fmicb.2025.1711088

**Published:** 2025-11-05

**Authors:** Mohammad Enamul Hoque Kayesh, Michinori Kohara, Kyoko Tsukiyama-Kohara

**Affiliations:** ^1^Department of Microbiology and Public Health, Faculty of Animal Science and Veterinary Medicine, Patuakhali Science and Technology University, Barishal, Bangladesh; ^2^Transboundary Animal Diseases Center, Joint Faculty of Veterinary Medicine, Kagoshima University, Kagoshima, Japan; ^3^Department of Microbiology and Cell Biology, Tokyo Metropolitan Institute of Medical Science, Tokyo, Japan

**Keywords:** West Nile virus, Toll-like receptors, Toll-like receptor agonists, innate immunity, immune evasion, vaccine development

## Abstract

The West Nile virus (WNV), an emerging neurotropic flavivirus and a leading cause of viral encephalitis worldwide, represents a significant public health threat owing to its neuroinvasive potential and the absence of a licensed human vaccine. Understanding the host immune response to WNV, particularly the role of Toll-like receptors (TLRs), is critical for elucidating viral pathogenesis and developing therapeutic strategies. TLRs are essential for the detection of viral components, initiation of innate immunity, and shaping of adaptive responses. Despite progress in research, no clinically approved WNV vaccine is currently available for humans, highlighting the urgent need for effective vaccine development. This review summarizes the current knowledge regarding the TLR-mediated immunity in WNV infection, with a focus on immune activation mechanisms and viral evasion strategies. Furthermore, we examine recent advances in vaccine development, emphasizing the potential of TLR agonists as adjuvants to enhance immunogenicity and protective efficacy.

## 1 Introduction

The West Nile virus (WNV) is an emerging mosquito-borne flavivirus that causes acute viral encephalitis with long-term neurological sequelae in humans and horses (Fulton et al., 2020; Saiz, 2020). Although most WNV infections are asymptomatic or mild, in some cases, severe neuroinvasive diseases, including meningitis, encephalitis, and acute flaccid paralysis develop, particularly in older or immunocompromised individuals (Sejvar, 2014; Gould et al., 2023). First identified in Uganda in 1937 (Smithburn et al., 1940) and introduced into the Western Hemisphere in 1999 (Colpitts et al., 2012), WNV is now widely distributed across Africa, Europe, Asia, and the Americas (Chancey et al., 2015). Transmission occurs primarily via *Culex* mosquitoes, with birds serving as amplifying hosts in the enzootic cycle, whereas humans and horses are incidental dead-end hosts (Chancey et al., 2015). Although ticks have been shown to transmit WNV in the laboratory, their role in the natural transmission and maintenance of the virus remains unclear (Abbassy et al., 1993; Hutcheson et al., 2005; Formosinho and Santos-Silva, 2006). In addition to the vector-borne transmission, alternative routes, such as blood transfusions, organ transplantation, and intrauterine transfer, have also been documented (Zeller and Schuffenecker, 2004).

West Nile virus belongs to genus *Flavivirus* in the *Flaviviridae* family, which also includes other medically important viruses, such as yellow fever virus, dengue virus, and Japanese encephalitis virus (Daep et al., 2014). The WNV genome contains a single-stranded, positive-sense RNA of approximately 11,000 nucleotides in length. It is translated into a single polyprotein that undergoes co- and post-translational cleavage to generate ten distinct proteins: three structural proteins — envelope (E), membrane (M), and nucleocapsid (C) — and seven non-structural (NS) proteins, including NS1, NS2A, NS2B, NS3, NS4A, NS4B, and NS5 (Anderson et al., 1999; Lanciotti et al., 1999). Non-structural proteins play key roles in viral transcription, translation, replication, maturation, and immune evasion (Diamond et al., 2009).

Although WNV was previously classified into two major lineages (Zeller and Schuffenecker, 2004), phylogenetic analyses have identified nine distinct lineages: 1a, 1b, 1c, 2, 3, 4, 7, and 8 (Koch et al., 2024). Among these, lineages 1a and 2 are most commonly associated with human disease. Lineage 1a is widely distributed across Africa, Europe, the Middle East, parts of Asia, Oceania, and the United States, whereas lineage 2, once confined only to sub-Saharan Africa, has recently emerged in Europe and established endemic transmission (Davis et al., 2024; Koch et al., 2024). Initially linked to sporadic outbreaks, the WNV has become a major cause of neurological diseases over recent decades, particularly in North America, where it led to severe conditions, such as meningitis and encephalitis (Kramer et al., 2007).

Currently, no licensed therapies or vaccines have been approved for WNV in humans, although several vaccines have been developed and approved for use in horses (El Garch et al., 2008; Kocabiyik et al., 2025). Therefore, safe and effective human vaccines are urgently needed. Various vaccine platforms, including live-attenuated, inactivated, nucleic acid-based, viral vector, and recombinant subunit vaccines, have been investigated, with several candidates demonstrating favorable immunogenicity and safety profiles in clinical trials (Saiz, 2020; Kocabiyik et al., 2025). Advanced adjuvant formulations offer a promising strategy for enhancing vaccine efficacy, particularly in the context of emerging or reemerging viral threats (Reed et al., 2013). Adjuvants enhance vaccine efficacy through multiple mechanisms, including promoting the maturation of antigen-presenting cells, enhancing T cell activation, and increasing the production of cytokines, multifunctional T cells, and antibodies (Zhao et al., 2023). Although aluminum salts (e.g., alum) are widely used, newer adjuvants, such as CpG ODN 1018, AS01, AS03, and AS04, have been incorporated into licensed vaccines (Iwasaki and Omer, 2020). Notably, Toll-like receptor (TLR) agonists have shown promise as adjuvants in vaccines against pathogens, including viruses such as hepatitis B virus, human papillomavirus, varicella zoster virus, and respiratory syncytial virus, thereby supporting their potential utility in future WNV vaccine development (Kayesh et al., 2023; Carter et al., 2025).

The innate immune response serves as the first line of defense against viral infections and plays a critical role in shaping disease outcomes and directing adaptive immunity (Diamond and Kanneganti, 2022). Recent advances have shed light on the complex interactions between viruses and innate immune pathways, including TLR signaling (Kawai et al., 2024). Elucidating these mechanisms is particularly important for understanding WNV pathogenesis and the development of targeted antiviral therapies (Lim et al., 2011). TLRs are pattern recognition receptors (PRRs) that play a key role in antiviral immunity by recognizing viral components, including viral nucleic acids and proteins, and triggering innate immune responses that regulate viral replication and shape the host’s defense mechanisms (Lester and Li, 2014).

Humans possess 10 TLRs (TLR1–TLR10), whereas mice have 12 TLRs (TLR1–TLR9 and TLR11–TLR13) (Kawasaki and Kawai, 2014). TLR1, TLR2, TLR4–TLR6, and TLR10 are expressed on the cell surface and primarily detect viral proteins, whereas TLR3, TLR7, TLR8, and TLR9 are localized intracellularly (mainly in the endoplasmic reticulum and endosomes) and recognize viral RNA and DNA (Alexopoulou et al., 2001; Diebold et al., 2004; Kawai and Akira, 2008; Chaturvedi and Pierce, 2009; Heim and Thimme, 2014; Lee et al., 2014). Upon activation, TLRs signal through adaptor proteins, most commonly MyD88, except for TLR3, which exclusively uses TRIF, to trigger downstream signaling pathways, leading to the production of proinflammatory cytokines, chemokines, and type I interferons (IFNs) (Medzhitov and Janeway, 2000; Akira et al., 2001; Lee and Kim, 2007; Mogensen, 2009; Fitzgerald and Kagan, 2020). These responses, essential for early viral recognition and adaptive immunity priming, determine infection outcomes (Lester and Li, 2014; Carty et al., 2021). TLRs are a double-edged sword, as although they are necessary for early pathogen recognition and the initiation of host defense, their dysregulation may lead to pathological immune responses instead of providing protection (Huang et al., 2008; Yokota et al., 2010; Modhiran et al., 2015; Kayesh et al., 2021). Therefore, thorough understanding of the involvement of TLRs in WNV infection is critical for the elucidation of immunopathogenetic mechanisms and development of effective therapeutic and preventive strategies. This review outlines the current knowledge regarding host TLR response to the WNV, highlights viral immune evasion mechanisms, and examines the potential of TLR-targeted approaches, particularly the use of TLR agonists as vaccine adjuvants, in advancing WNV vaccine development.

## 2 Innate immune response to West Nile virus infection

The innate immune response serves as the first line of defense against invading pathogens and plays a crucial role in preventing infections (Marshall et al., 2018). Pattern recognition receptors (PRRs), including TLRs, RIG-I-like receptors (RLRs), nucleotide-binding oligomerization domain (NOD)-like receptors (NLRs), protein kinase R, oligoadenylate synthetase (OAS), absent in melanoma-2, C-type lectin receptors, and cyclic GMP-AMP synthase (cGAS)–stimulator of interferon genes (STING) pathway, play critical roles in initiating and regulating the innate immune response against WNV infection (Behari et al., 2024). As with many other RNA and DNA viruses, type I interferons (IFN-α/β) play a critical role in controlling WNV infection and limiting disease development through the induction of IFN-stimulated genes (ISGs) (Samuel and Diamond, 2005; Daffis et al., 2011; Lazear et al., 2011). Several protein products of ISGs and their receptors, including IRF1, C6orf150, HPSE, RIG-I, MDA5, and IFITM3, possess direct antiviral activity against WNV, underscoring the complexity and breadth of IFN-mediated antiviral defenses (Schoggins et al., 2011). High-throughput overexpression screening has identified both broadly acting and WNV-specific ISGs, revealing a multifaceted network of antiviral effectors (Schoggins et al., 2011). Among these ISGs, the *Oas1b* gene was shown to play a critical role in controlling WNV infection in mice. Green et al. (2017) demonstrated that *Oas1b* influenced host susceptibility, disease severity, and tissue-specific gene expression, thereby contributing to OAS1B-dependent and independent antiviral mechanisms. These findings suggest that *Oas1b* is a key genetic determinant of resistance to WNV (Green et al., 2017).

Although it is traditionally associated with DNA sensing, cyclic GMP-AMP synthase (cGAS) also contributes to the immune response against RNA viruses, including WNV, via a STING-dependent IRF3-mediated pathway that functions independently of the canonical IFN/STAT1 signaling (Schoggins et al., 2015). Mice lacking cGAS are significantly more susceptible to lethal WNV infection, with an elevated viral load observed in bone marrow-derived macrophages compared to that in macrophages from wild-type counterparts, which demonstrates a protective role for cGAS in WNV control (Schoggins et al., 2015).

Early detection of WNV is mediated by PRRs, particularly RLRs such as RIG-I and MDA5, which detect cytosolic viral RNA. Recent studies highlight the pivotal role of RIG-I in initiating innate immune responses by detecting cytoplasmic antigenomic negative-sense viral RNA (-vRNA). Although flaviviruses typically conceal -vRNA within membrane-bound replication compartments to evade immune detection, a small amount can escape—likely facilitated by viral capsid proteins—during the later stages of infection, triggering antiviral signaling via RIG-I activation (Andino and Darling, 2025; Genoyer et al., 2025). These RLR-mediated receptors are critical for limiting viral replication and promoting host survival (Fredericksen and Gale, 2006). MDA5 plays a protective role during WNV infection by promoting antiviral immunity in the central nervous system (CNS). Mice deficient in MDA5 show increased susceptibility to WNV, characterized by increased viral loads in the CNS and impaired CD8^+^ T cell responses, despite modest effects on peripheral viral control and no direct impact on neuronal infection (Lazear et al., 2013). RIG-I is critical for the early sensing of WNV, and together with MDA5, it drives the robust induction of innate immune genes. Loss of either receptor alone impairs immune signaling and increases mortality; however, the combined deletion of RIG-I and MDA5 results in the complete failure of antiviral gene expression and severe disease outcomes comparable to those observed in MAVS-deficient mice. These findings highlight the non-redundant, complementary roles of RIG-I and MDA5, which detect distinct pathogen-associated molecular patterns during different phases of viral replication (Errett et al., 2013). These RLRs signal through the adaptor protein IPS-1 (also known as MAVS), which is essential for activating downstream pathways and production of type I IFN and proinflammatory cytokines (Kumar et al., 2006). IPS-1 deficiency results in uncontrolled viral replication, impaired cytokine responses, and increased susceptibility to WNV infection (Suthar et al., 2010). Similarly, transcription factor ELF4 enhances antiviral immunity through the MAVS-TBK1 signaling (You et al., 2013), and IRF3 is indispensable for limiting viral replication in peripheral and CNS tissues (Daffis et al., 2007).

In addition to RLRs, the phosphatidylinositol 3-kinase (PI3K) signaling pathway also contributes to the antiviral defense against WNV. PI3K plays a critical role in the regulation of type I IFN responses by promoting IRF7 nuclear translocation, which is essential for IFN production. Pharmacological inhibition of PI3K significantly increases viral replication and impairs IRF7 activation, underscoring the importance of this pathway in mounting effective innate immune responses during WNV infection (Wang et al., 2017).

Mice lacking both MyD88 and TRIF— the two key adaptor proteins in TLR signaling — display an even greater susceptibility to WNV infection than mice lacking either adaptor alone, indicating their cooperative roles in host defense. These double knockout mice showed markedly reduced levels of innate immune cytokines, further emphasizing the importance of TLR-mediated pathways in coordinating effective antiviral responses (Sabouri et al., 2014). However, the absence of individual TLRs, such as TLR9 or TLR4, did not significantly alter the susceptibility to WNV infection, suggesting that these receptors are not essential on their own in this context (Sabouri et al., 2014).

While TLR3 recognizes WNV-derived dsRNA, there is no evidence that it directly mediates viral entry. TLRs play diverse and sometimes contrasting roles in WNV pathogenesis. TLR3 limits viral replication in neurons, and protects against neuroinvasive diseases (Daffis et al., 2008), but its function is context-dependent. TLR3 expression is downregulated in macrophages from young individuals via a STAT1-dependent mechanism during WNV infection, but this regulation is impaired in the elderly, leading to elevated TLR3 levels and increased cytokine production. This dysregulation may contribute to blood-brain barrier (BBB) permeability, contributing to the increased severity of WNV infections in aged populations (Kong et al., 2008). Furthermore, TLR3 may not significantly prevent viral entry into the brain, but instead regulates inflammation within the CNS. NS1 immunization has been shown to reduce neuroinflammation, even in TLR3-deficient mice, suggesting TLR3 contribution is more immunomodulatory rather than directly antiviral in this context (Patel et al., 2019). In support of this notion, TLR3 was also shown to promote WNV neuroinvasion by enhancing inflammation-induced BBB disruption, highlighting its dual role in peripheral defense and CNS pathology (Wang et al., 2004).

TLR7 also plays a dual role in WNV infection. Although TLR7- and MyD88-deficient mice are highly susceptible to mutant WNV infection, suggesting a protective function (Xie et al., 2013), TLR7 has also been implicated in facilitating viral dissemination. In murine models, TLR7 promotes IL-23-mediated immune cells recruitment to infected tissues, and limits viral spread and disease severity (Town et al., 2009). At the cellular level, TLR7 enhances antiviral defenses in keratinocytes through the increased production of IFN-α and inflammatory cytokines (Welte et al., 2009). However, the same TLR7-driven responses may also promote WNV spread from the skin to peripheral organs, contributing to systemic infection (Welte et al., 2009). Furthermore, TLR8 may modulate TLR7-mediated immunity, potentially enhancing WNV pathogenesis by suppressing antiviral responses via interaction with SOCS-1, a negative regulator of IFN signaling (Paul et al., 2015).

MyD88 serves as a central adaptor protein in TLR signaling, mediating downstream inflammatory responses through recruitment of IRAK family kinases and activation of key transcription factors, such as NF-κB and AP-1 (Deguine and Barton, 2014). During WNV infection, MyD88 plays a critical role in limiting viral replication in specific cell types and supports the chemokine-driven recruitment of immune cells to the CNS. Although systemic type I IFN responses are largely preserved in MyD88-deficient mice, they show elevated viral loads in the brain and increased mortality, highlighting the importance of theMyD88-mediated inflammatory pathways in CNS protection (Szretter et al., 2010).

Polymorphisms in TLR genes have been shown to influence host susceptibility and clinical outcomes in both DNA and RNA virus infections (Carty and Bowie, 2010; Medvedev, 2013). Many studies have reported the associations between single-nucleotide polymorphisms (SNPs) of the specific TLR genes and disease severity in RNA virus infections, including those caused by SARS-CoV-2 (Alhabibi et al., 2023; Parsania et al., 2024), HIV (Oh et al., 2009), HCV (Du et al., 2023b), dengue virus (Alagarasu et al., 2015), Zika virus (Santos et al., 2023), and Japanese encephalitis virus (JEV) (Biyani et al., 2015). For example, the TLR3 Leu412Phe polymorphism has been found at a higher frequency in patients with JEV compared to healthy controls, suggesting a possible role in neurotropic flavivirus infections (Biyani et al., 2015). However, to date, no direct association between TLR polymorphisms and clinical outcomes in human WNV infection has been established, highlighting an important gap in our understanding that warrants further investigation.

**FIGURE 1:**
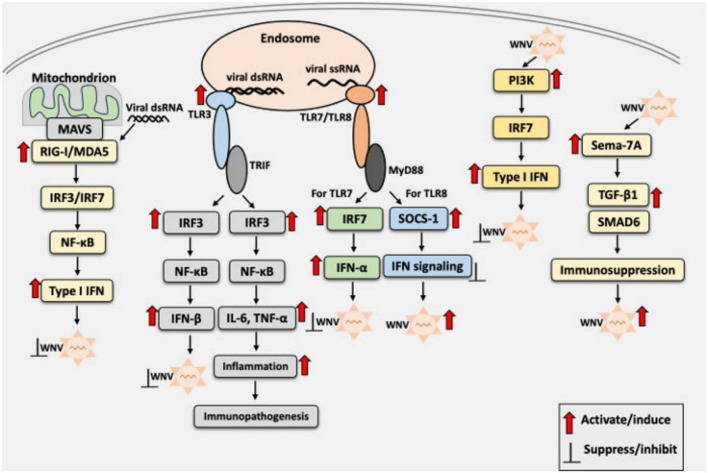
Schematic overview of the innate immune response to West Nile Virus (WNV) infection. Upon WNV entry into the host cells, viral RNAs are recognized by the host pattern recognition receptors (PRRs), including RIG-I, MDA5 (cytosolic sensors), and TLR3 or TLR7 (endosomal sensors), as appropriate. Recognition by these receptors initiates signaling via IRF3/7 and NF-κB, leading to the production of type I interferons (IFN-α/β), proinflammatory cytokines, and chemokines. IFNs restrict viral replication, whereas excessive TLR3-mediated inflammation may drive immunopathology. TLR7 acts as a double-edged sword, promoting antiviral immunity via the MyD88-dependent signaling. However, TLR8 suppresses TLR7 responses through SOCS1, potentially promoting pathogenesis. Red arrows indicate activation of innate immune signaling by WNV or its components, enhancing or reducing viral replication. Black blunt arrows indicate suppression or inhibition of the host innate immune response or WNV replication, as appropriate. IFN, interferon; IL-6, interleukin-6; IRF3, interferon regulatory factor 3; IRF7, interferon regulatory factor 7; ISG, interferon-stimulated gene; MyD88, myeloid differentiation primary response 88; NF-κB, nuclear factor kappa-light-chain-enhancer of activated B cells; SOCS1, suppressor of cytokine signaling 1; TNF-α, tumor necrosis factor-alpha; TRIF; TIR domain-containing adaptor-inducing IFN-β.

In addition to classical innate immune pathways, several host factors modulate WNV pathogenesis. For example, Pellino 1 (Peli1), an E3 ubiquitin ligase and adaptor protein involved in TLR signaling (Choi et al., 2006), facilitates WNV entry and replication in neurons, microglia, and macrophages (Luo et al., 2018). Peli1 enhances pro-inflammatory cytokine and chemokine production in the CNS, contributing to neuroinflammation and disease severity. *Peli1*-deficient mice showed improved survival and reduced viral loads, suggesting a pathogenic role in WNV infection (Luo et al., 2018). Moreover, inhibition of Peli1 has been shown to reduce disease in Zika virus infection, suggesting its therapeutic potential across flaviviruses (Luo et al., 2020). Another host factor that contributes to the pathogenesis is semaphorin 7A (Sema7A), which facilitates viral replication, increases blood–brain barrier permeability, and enhances expression levels of TGF-β1 and SMAD6, both of which are associated with immunomodulation and neuroinflammation. Mice lacking *Sema7A* show enhanced survival and reduced viral burden, underscoring Sema7A function as a proviral factor during WNV infection (Sultana et al., 2012). Together, these findings emphasize the complexity of the innate immune landscape in WNV infection, where both antiviral and proviral host factors, along with finely tuned signaling pathways, determine disease outcomes and highlight potential targets for therapeutic intervention. A schematic overview of the innate immune response to WNV infection, including the roles of PRRs, IFNs, and inflammatory signaling, is presented in Figure 1.

## 3 Inhibition of innate immune response by WNV infection

West Nile virus employs multiple strategies to subvert host innate immunity, thereby facilitating viral replication, dissemination, and persistence. A major component of this evasion strategy is the suppression of PRR signaling and downstream IFN responses. WNV non-structural protein 1 (NS1), a multifunctional glycoprotein localized intracellularly and in the plasma membrane, plays a pivotal role in immune evasion. Secreted NS1 disrupts TLR signaling pathways by inhibiting the TLR3, TLR4, and TLR7 pathways, leading to reduced cytokine production in macrophages and dendritic cells both *in vitro* and *in vivo* (Crook et al., 2014). Wilson et al. (2008) further demonstrated that WNV NS1 disrupts TLR3 signaling by inhibiting nuclear translocation of IRF3 and NF-κB, thereby preventing the transcriptional activation of the IFN-β promoter and TLR3-dependent interleukin-6 (IL-6) production (Wilson et al., 2008). This interference effectively suppresses the innate immune response, contributing to viral evasion of host defenses (Wilson et al., 2008). However, no evidence of WNV non-structural proteins inhibiting IRF3 activation was reported, suggesting that the interaction between the WNV and IRF3 may be context-dependent (Fredericksen and Gale, 2006).

In addition to modulating TLR signaling, NS1 facilitates immune evasion by interacting with the complement system. NS1 binds to the complement regulatory protein factor H, promoting C3b cleavage and inactivation of the alternative complement pathway (Zipfel et al., 2002). Consequently, NS1 inhibits complement activation both in solution and on cell surfaces, reducing the deposition of C3 fragments and C5b–9 membrane attack complexes, thereby limiting immune recognition of infected cells (Chung et al., 2006).

Zhang et al. (2017) demonstrated that WNV NS1 interacts with RIG-I and MDA5, promoting their proteasomal degradation and blocking the K63-linked polyubiquitination of RIG-I, which is an essential step for downstream signaling. As a result, NS1 inhibits IRF3 phosphorylation and nuclear translocation, impairing IFN-β production and dampening the antiviral response (Zhang et al., 2017). WNV infection also inhibits poly(I:C)-induced IRF3 activation and subsequent IFN-β transcription (Scholle and Mason, 2005).

In addition to NS1, other WNV non-structural proteins, including NS2A, NS2B, NS3, NS4A, and NS4B, contribute significantly to immune evasion (Liu et al., 2005). Specifically, these proteins from the WNV Kunjin strain have been shown to block IFN-α-induced STAT2 activation, inhibiting JAK-STAT signaling and the induction of ISGs (Liu et al., 2005). In addition, the NS5 protein of certain flaviviruses interferes with IFN signaling by inhibiting STAT1 phosphorylation or promoting STAT2 degradation. In the context of the WNV, NS5 disrupts TLR3-mediated type I IFN production (Laurent-Rolle et al., 2010). Mutational analysis further highlighted the immunomodulatory role of WNV non-structural proteins. Mutations in NS4B altered TLR expression profiles, which may indirectly influence RLR-mediated responses, indicating a complex interplay between these innate immune pathways during WNV infection (Xie et al., 2015).

West Nile virus structural proteins also contribute to immune evasion. The envelope protein suppresses double-stranded RNA-induced cytokine production in murine macrophages via a TLR3-independent mechanism involving receptor-interacting protein 1 (Arjona et al., 2007). Furthermore, the WNV impairs functions of dendritic cells (DCs), key players bridging innate and adaptive immunity (Zimmerman et al., 2019). WNV-infected DCs have reduced expression of proinflammatory cytokines (IL-6, granulocyte-macrophage colony-stimulating factor, CCL3, CCL5, and CXCL9) and T cell modulatory cytokines (IL-4, IL-12, and IL-15), ultimately weakening the early immune response and T cell activation (Zimmerman et al., 2019).

**FIGURE 2:**
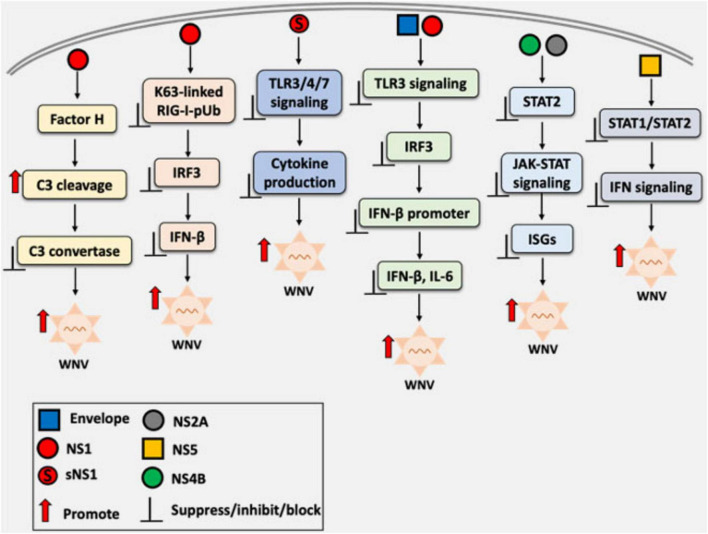
Overview of West Nile virus (WNV)-mediated immune evasion strategies through the modulation of host innate immune signaling. Both structural and non-structural proteins interfere with pattern recognition receptor (PRR) signaling pathways, including Toll-like receptors (TLRs), RIG-I-like receptors (RLRs), and other components of the innate immune response. Non-structural protein 1 (NS1), including its secretory form (sNS1), appears to be a key protein that disrupts critical steps in the interferon (IFN) response cascade, such as inhibiting interferon regulatory factor 3 (IRF3) activation and interfering with interferon-β promoter activation. The overall effect is attenuation of antiviral responses and the resulting increase in viral replication.

The immune evasion capacity determines the virulence of WNV strains. The lineage II MAD78 strain is highly sensitive to type I IFNs and unable to antagonize IFN-induced JAK-STAT signaling, unlike the more pathogenic lineage I TX02 strain. These findings underscore the importance of IFN antagonism in determining WNV replication, fitness, and virulence (Keller et al., 2006). An overview of the WNV-mediated modulation of host innate immune responses, highlighting how structural and non-structural proteins, including NS1, disrupt interferon signaling, is illustrated in Figure 2.

## 4 Agonists of TLRs as adjuvants in WNV vaccine development

Toll-like receptor agonists have emerged as promising vaccine adjuvants for enhancing immunity against viral infections, including those caused by flaviviruses, by stimulating robust innate and adaptive immune responses (Kayesh et al., 2023). TLR4 agonist adjuvant significantly enhanced the immunogenicity and protective efficacy of a promising clinical-stage recombinant WNV E-protein vaccine WN-80E (Van Hoeven et al., 2016). The formulation of WN-80E with TLR4 agonists in either a stable oil-in-water emulsion or aluminum hydroxide robustly protected C57BL/6 mice after a single low-dose immunization, which correlated with Th1-skewed immune responses and undetectable serum WNV loads (Van Hoeven et al., 2016). These findings underscore the potential of TLR4-based adjuvants to improve WNV vaccine performance and support their continued development for use in rapid-response vaccination strategies. Building on this work, Van Hoeven et al. (2018) investigated advanced adjuvant formulations by combining WNV recombinant antigens with the potent TLR4 agonist SLA, saponin QS21, or a combination thereof delivered in liposomal formulations. These formulations induced strong adaptive immune responses and high neutralizing antibody titers after a single immunization in both mouse and hamster models. Importantly, this immune response conferred long-lasting immunity and protection against the WNV challenge (Van Hoeven et al., 2018). Notably, adjuvants based on TLR4 agonists have been approved for use in several viral vaccines, including those against hepatitis B virus, human papillomavirus, varicella zoster virus, and respiratory syncytial virus (Carter et al., 2025).

TLR5 agonists have gained attention because of their ability to enhance immune responses by activating innate signaling pathways, highlighting their potential as effective vaccine adjuvants (Hajam et al., 2017). For example, modified flagellin, a TLR5 agonist fused to domain III of the WNV E protein, enhanced both the innate and adaptive immune responses, providing strong protection without the need for additional adjuvants (McDonald et al., 2007). However, age-related differences in vaccine efficacy remain a critical consideration. For example, aged mice (21–22 months old) were susceptible to infection with the attenuated WNV NS4B-P38G mutant that was otherwise safe and immunogenic in young mice (Xie et al., 2016). In aged mice, NS4B-P38G infection resulted in elevated levels of inflammatory cytokines and IL-10, delayed γδ T cell expansion, and reduced antibody and WNV-specific T cell responses. These defects were attributed to age-related dysregulation of TLR7 signaling (Xie et al., 2016). Notably, administration of R848, a synthetic TLR7 agonist (Okuzumi et al., 2021), enhanced immune responses in aged mice vaccinated with the NS 4B-P38G mutant, by restoring DC function and promoting γδ T cell and regulatory T cell expansion (Xie et al., 2016), suggesting important implications for the use of a TLR7 agonist in the context of a WNV vaccine, particularly for the elderly.

TLR9 agonists, such as CpG oligodeoxynucleotides, have shown considerable promise as adjuvants for WNV vaccines by enhancing both humoral and cell-mediated immune responses. When delivered via surface-modified nanoparticles (NPs) carrying the WNV envelope protein, these agonists elicited strong Th1-biased immunity and provided superior protection compared to that afforded by conventional alum-based adjuvants (Demento et al., 2010). These findings indicate that other TLR agonists may be incorporated into NP-based vaccine platforms to further optimize immune responses against the WNV and related flaviviruses. A careful selection of TLR agonists as adjuvants is essential for the development of effective and well-tolerated WNV vaccines. Further studies are warranted to evaluate the use of individual TLR agonists or their combinations to optimize immune responses and improve the efficacy of WNV vaccine formulations.

## 5 WNV vaccines in preclinical development

Several WNV vaccine candidates are currently undergoing preclinical evaluations for the ability to induce strong and protective immune responses. These include subunit, DNA, viral vector-based, and live-attenuated vaccines, most of which target key viral components, such as the envelope (E) protein (Kaiser and Barrett, 2019). A promising approach involves a plasmid DNA vaccine encoding the ectodomain of the WNV E protein, formulated into NPs using mannose-modified linear polyethyleneimine. In a murine model, when followed by a heterologous boost with a recombinant E protein, this vaccine elicited robust neutralizing antibody and T cell responses, providing effective protection against the lethal WNV challenge (De Filette et al., 2014b). Additionally, DNA vaccine-generated subviral particles expanded the WNV-E-specific T-cell repertoire in Balb/c mice, demonstrating a potent and targeted cellular immune response (De Filette et al., 2014a). Similarly, another DNA vaccine expressing only the ectodomain of the WNV E protein induced strong T-cell responses and neutralizing antibodies in mice and conferred full protection against a lethal challenge (Schneeweiss et al., 2011). Immunogenicity was further enhanced by a recombinant protein boost, supporting the role of E protein as a key antigen in DNA vaccine development (Schneeweiss et al., 2011).

A recombinant subunit vaccine based on the WNV E protein (WN-80E), with or without NS1, and formulated with the GPI-0100 adjuvant, also demonstrated strong immunogenicity. It conferred protection to young, aged, and immunocompromised hamsters, highlighting the potential of this vaccine to achieve a broader population coverage (Siirin et al., 2008). In another strategy, an intranasally administered vesicular stomatitis virus-based vaccine expressing the WNV E protein, administered at a dose of 10^5^ PFU per mouse with a booster on day 21, induced both humoral and cellular immunity and protected mice against the WNV lethal challenge (Iyer et al., 2009).

Recombinant WNV E and domain III proteins produced in insect larvae also showed strong immunogenicity, eliciting high titers of neutralizing antibodies and providing complete protection against challenge with neurovirulent WNV NY99 in mice (Alonso-Padilla et al., 2011). These results support the use of insect-derived antigens as cost-effective subunit vaccines. The conjugation of WNV E protein domain III to bacteriophage AP205 virus-like particles significantly enhanced immunogenicity, providing robust neutralizing antibody responses and full protection after three doses (Spohn et al., 2010). The combination of efficacy, safety, and low production costs make this conjugate vaccine platform particularly promising. Similarly, a plant-based approach using domain III of the WNV E protein expressed in *Nicotiana benthamiana* elicited a robust systemic immune response in mice following subcutaneous immunization, demonstrating its potential as an economical and scalable WNV vaccine candidate (He et al., 2014).

Immunogenicity of a subunit vaccine candidate consisting of the recombinant truncated WNV E protein (rWNV-80E) formulated with alum and CpG adjuvants was evaluated in C57BL/6 mice (Du et al., 2023a). The vaccine elicited strong humoral and cellular immune responses, including high titers of neutralizing antibodies and T cell-derived IFN-γ and TNF-α, indicating its potential as a promising WNV vaccine candidate for further investigation (Du et al., 2023a).

A plant-produced virus-like particle (VLP) displaying WNV E protein domain III was shown to induce potent neutralizing antibody and antigen-specific cellular immune responses in mice, while also reducing the risk of antibody-dependent enhancement, a concern commonly associated with severe dengue or Zika virus infections (Sun et al., 2023). Similarly, immunization with the wild-type WNV E protein provided complete protection against viral challenge in mice (Weiß et al., 2023). In contrast, modified antigens either incorporating a mutated fusion loop or consisting solely of domain III provided only partial protection. However, these modified constructs significantly reduced serological cross-reactivity with heterologous flaviviruses, such as dengue and Zika, highlighting a promising strategy for enhancing WNV vaccine specificity (Weiß et al., 2023). Additionally, a recent preclinical study by Vorovitch et al. (2024) evaluated an inactivated whole-virion WNV vaccine based on the SHUA strain, which achieved 100% seroconversion and conferred complete protection against a lethal viral challenge to mice, demonstrating strong potential as a safe and effective vaccine candidate for preventing severe WNV infections (Vorovitch et al., 2024). Collectively, these preclinical studies illustrate the progress and diversity of WNV vaccine platforms in development, emphasizing how rational antigen design and novel delivery systems can enhance immunogenicity, improve safety, and reduce cross-reactivity with related flaviviruses.

## 6 WNV vaccines in clinical development

Vaccination remains the most effective strategy for preventing infectious diseases, including those caused by flaviviruses such as WNV (Gould et al., 2023). Currently, several vaccine candidates are at various phases of clinical development. ChimeriVax-WN02, a live attenuated chimeric vaccine, has shown considerable promise. In a phase I clinical trial, it elicited robust immune responses after a single dose, supporting its potential as a candidate for the prevention of WNV disease (Monath et al., 2006). In a subsequent Phase II randomized, double-blind, placebo-controlled trial, ChimeriVax-WN02 demonstrated high immunogenicity and a favorable safety profile across all age groups, with seroconversion rates exceeding 96%. The highest dose induced stronger antibody responses and reduced viremia (Biedenbender et al., 2011). A separate phase II study confirmed its immunogenicity and safety, reinforcing the rationale for continued clinical development (Gould et al., 2023).

The National Institutes of Health has developed a recombinant live-attenuated vaccine candidate, rWN/DEN4Δ30, in which genes encoding the premembrane (prM) and envelope (E) proteins of the WNV NY99 strain replaced those of the live-attenuated dengue virus serotype 4 (rDEN4Δ30) backbone (Pletnev et al., 2002). In phase I clinical trials, the rWN/DEN4Δ30 chimeric vaccine was well-tolerated and immunogenic, with seroconversion rates ranging from 74% to 89%, depending on the dose and vaccination schedule. These findings support continued clinical development, particularly for use with older adults (Durbin et al., 2013; Pierce et al., 2017). Similarly, in a phase I trial, HydroVax-001, a hydrogen peroxide–inactivated WNV vaccine adjuvanted with aluminum hydroxide, was found to be safe and well-tolerated, with no serious side effects. A dose of 1 μg elicited a limited immune response, whereas a dose of 4 μg elicited stronger responses, with up to 75% of participants developing antibodies, depending on the assay used (Woods et al., 2019). Building on these results, a subsequent phase 1 trial of HydroVax-001B was launched on 24 February 2025, to evaluate higher doses of 4 and 10 μg (ClinicalTrials.gov ID: NCT06745921).

In a phase 1 open-label trial, a DNA vaccine encoding WNV prM and E proteins was found to be safe and well-tolerated, with no significant adverse events. Neutralizing antibody and T cell responses were observed in most participants who completed the three-dose regimen (Martin et al., 2007). A subsequent phase 1 trial confirmed these findings and further demonstrated immunogenicity in adults aged 51–65 years, a population that is typically less responsive to conventional vaccines (Ledgerwood et al., 2011). Collectively, these early phase clinical trials of WNV vaccine candidates, including live-attenuated, inactivated, chimeric, and DNA-based platforms, have shown promising safety and immunogenicity profiles. However, the limited follow-up duration of most studies restricts our understanding of the durability of vaccine-induced protection. Additionally, the absence of a universally accepted immune correlate of protection against WNV complicates the interpretation of immunogenicity data. Continued clinical development is essential to determine the most effective strategies for protecting diverse high-risk populations. An overview of WNV vaccine candidates currently in clinical development is presented in Table 1.

**TABLE 1:** Summary of West Nile virus (WNV) vaccines under clinical development.

Vaccine name	Vaccine type	Vaccine formulation/antigen	Developer/sponsor/manufacturer	Clinical phase and trial duration	Participants enrolled	Dosage and route	Target age group	Strengths	Limitations	References/clinical trial number
ChimeriVax-WN02	Live, attenuated chimeric vaccine	prM/E from WNV NY99 inserted into YFV 17D backbone	Sanofi Pasteur	Phase 2	112 and 96	Single dose, SC	18–50 years	Highly immunogenic, high seroconversion rates (> 96%), no severe adverse effect	No data on children/adolescents; all subjects seronegative; ADE not evaluated	(Lanciotti et al., 1999; Monath et al., 2006; Biedenbender et al., 2011); NCT00442169
ChimeriVax-WN02	Live, attenuated chimeric vaccine	prM/E from WNV NY99 inserted into YFV 17D backbone	Sanofi Pasteur	Phase 2	479	Single dose of ∼ 4 × 10^3^ to 10^5^ PFU, SC	≥ 50 years of age	Highly immunogenic, 92%–95% seroconversion	Same as above	(Gould et al., 2023); NCT00746798
ChimeriVax-WN02	Live, attenuated chimeric vaccine	prM/E from WNV NY99 inserted into YFV 17D backbone	Sanofi Pasteur	Phase 1	80	Single dose (5.0/3.0 log*10* PFU)	18–40 years	Robust immunogenicity; 100% seroconversion	Same as above	Monath et al., 2006
rWN/DEN4Δ30	Live, attenuated chimeric vaccine	prM/E from WNV NY99 strain inserted into DENV-4 backbone (rDEN4Δ30)	National Institute of Allergy and Infectious Diseases (NIAID)	Phase 1	56	Single dose of 10^3^ or 10*4* PFU, SC	18–50 years	Well-tolerated, immunogenic, 74% (10^3^ PFU), 75% seroconversion	Same as above	(Durbin et al., 2013); NCT00094718
rWN/DEN4Δ30	Live, attenuated chimeric vaccine	prM/E from WNV NY99 strain inserted into DENV-4 backbone (rDEN4Δ30)	NIAID	Phase 1	26	Two doses of 10*5* PFU, 6 months apart, SC	18–50 years	Well-tolerated, immunogenic; 55% seroconversion (after single dose); 89% after 2^nd^ dose	Same as above	(Durbin et al., 2013); NCT00537147
rWN/DEN4Δ30	Live, attenuated chimeric vaccine	prM/E from WNV NY99 strain inserted into DENV-4 backbone (rDEN4Δ30)	NIAID	Phase 1	28	Two-dose regimen of 10*4* PFU, 6 months apart, SC	50–65 years	Well-tolerated and immunogenic	Same as above	NCT02186626
HydroVax-001 WNV vaccine	Inactivated WNV vaccine	Whole inactivated virion (WNV-Kunjin strain)	NIAID	Phase 1	96	1 or 4 μg; two IM doses, 28 days apart	18–49 years of age	Safe and well-tolerated	Same as above	(Woods et al., 2019); NCT02337868
HydroVax-001B	Inactivated WNV vaccine	Whole inactivated virion (WNV-Kunjin strain)	NIAID	Phase 1	30	4 or 10 μg; IM on Days 1, 29 and 181	18–49 years of age	Ongoing, no data released yet	Ongoing, no data released yet	NCT06745921
VRC-WNVDNA017-00-VP	Recombinant DNA vaccine	Plasmid DNA containing WNV prM/E genes (NY99 strain)	NIAID	Phase 1	15	Three-dose regimen, (Day 0, Day 28, Day 56)	18–50 years	Safe and well-tolerated; Highly immunogenic and 100% subjects produced neutralizing antibodies	All subjects seronegative; ADE not evaluated	(Martin et al., 2007); NCT00106769
VRC-WNVDNA020-00-VP	Recombinant DNA vaccine	Plasmid DNA containing WNV prM/E proteins	NIAID	Phase 1	30	Three-dose regimen (Day 0, Day 28, Day 56)	18–65 years	Safe and well-tolerated	Same as above	(Ledgerwood et al., 2011); NCT00300417
HBV-002/WN-80E	Recombinant subunit vaccine	Recombinant WNV E protein	Hawaii Biotech, Inc.	Phase 1	25	Three-dose regimen (5 or 15 or 50 μg); 1 month apart	18–45 years	Unpublished	Unpublished	NCT00707642

## 7 Discussion

Although much of our understanding of the TLR-mediated antiviral responses is derived from knockout mouse models, data on the functional role of TLRs in human viral immunity remain limited. However, several key insights have emerged. For example, human TLR3 has been implicated in the neuroprotection against herpes simplex virus 1 infection, with loss-of-function mutations in the corresponding gene associated with susceptibility to herpes simplex encephalitis (Zhang et al., 2007).

In the context of WNV infection, TLR3 appears to play a similarly context-dependent and tissue-specific role. Although *in vitro* studies using HEK293 cells have shown a minimal impact of TLR3 on viral replication or immune signaling (Chugh et al., 2014), *in vivo* data suggested an important role of TLR3. TLR3-deficient mice had an elevated viral burden in the brain and increased mortality despite normal peripheral interferon responses, highlighting a neuroprotective rather than systemic antiviral role (Daffis et al., 2008). Interestingly, TLR3 may not prevent viral entry into the CNS but instead likely modulates the inflammatory response once the infection is established (Patel et al., 2019). Moreover, WNV induces a largely TLR3-independent miRNA response, suggesting that other innate sensors may also regulate antiviral gene expression (Chugh et al., 2014).

The observation that TLR3 supports germinal center formation and long-lived plasma cell generation following vaccination with the RepliVAX WN platform further highlights its relevance for durable humoral immunity (Xia et al., 2013). These findings have important implications for vaccine design. TLR agonists are already used in licensed viral vaccines, so they may enhance the WNV vaccine efficacy by boosting long-term antibody responses. For example, TLR7/8 agonist INI-4001 has shown promise in preclinical models of the Powassan virus — a neuroinvasive flavivirus related to WNV — and may hold potential for WNV VLP-based vaccines by promoting strong innate activation and adaptive priming (Crawford et al., 2025). Although INI-4001 targets both TLR7 and TLR8, its design and dosing could potentially favor TLR7-driven responses, especially when used in a vaccine formulation where this balance is optimized (Kayesh et al., 2025). Despite these advances, WNV vaccine development continues to face significant challenges, including sporadic outbreaks, limited case numbers for efficacy trials, difficulty in distinguishing between vaccine-induced and natural immunity, and underrepresentation of high-risk populations. These factors limit broader clinical application of the tested vaccine preparations and regulatory progress (Gould et al., 2023).

In addition to adjuvants, some WNV vaccine platforms intrinsically activate the innate immune pathways. For instance, the RepliVAX WN vaccine includes built-in PAMPs and depends on both MyD88 and TLR3 signaling for optimal B cell activation and antibody longevity (Xia et al., 2013). This suggests that rational vaccine design can leverage innate sensing pathways to enhance protective immunity even in the absence of external adjuvants. In addition, RIG-I agonists, such as 5’-pppRNA, induce potent IFN-independent antiviral states and may offer complementary or synergistic benefits alongside TLR-based interventions (Goulet et al., 2013).

Finally, alternative immunotherapeutic strategies are also explored. A humanized, plant-derived monoclonal antibody targeting the WNV envelope protein (Hu-E16) showed protective efficacy in mice, even when administered up to 4 days post-infection, offering a proof-of-concept for passive immunotherapy approaches (Lai et al., 2010). Together, these findings underscore the importance of innate immune sensing not only in the early control of WNV infection but also in shaping the quality and durability of adaptive responses. Understanding how to manipulate TLR signaling in a controlled manner is essential for advancing both prophylactic and therapeutic strategies against WNV and related flaviviruses.

## 8 Limitations and future directions

Despite advances in understanding TLR-mediated sensing of WNV and current vaccine strategies, key gaps remain. The functional overlap and tissue-specific expression of TLRs complicate efforts to define their distinct roles in WNV pathogenesis, and most insights rely on murine models that may not fully reflect human immunity. Future studies should focus on TLR signaling in human primary cells, especially in the context of neuroinvasion.

Although TLR polymorphisms are linked to disease severity in other RNA virus infections, such associations remain unexplored for WNV. Genetic studies in endemic regions, coupled with functional analyses of TLR variants, are needed to clarify their role in host susceptibility. Vaccine development may also benefit from TLR-based adjuvants to enhance immune responses. A deeper understanding of viral evasion of TLR pathways will be critical for guiding next-generation vaccine and therapeutic strategies.

## 9 Conclusion

West Nile virus remains a significant global health threat because of its neuroinvasive potential, severe sequelae, and the absence of an approved human vaccine. Understanding the immune mechanisms, particularly the role of TLRs, is essential for deciphering WNV pathogenesis and guiding therapeutic development. TLRs serve as key mediators of antiviral defense by initiating and shaping immune responses, although the WNV has evolved strategies to evade the detection by TLRs. Advances in our understanding of TLR-mediated immunity offer promising avenues for vaccine development, especially by using TLR agonists as adjuvants to enhance the protective efficacy of vaccines. Continued research into these pathways is crucial for the development of effective vaccines and targeted interventions against WNV.

## References

[B1] Abbassy M. Osman M. Marzouk A. (1993). West nile virus (Flaviviridae:flavivirus) in experimentally infected Argas ticks (Acari:argasidae). *Am. J. Trop. Med. Hyg.* 48 726–737. 10.4269/ajtmh.1993.48.726 8517492

[B2] Akira S. Takeda K. Kaisho T. (2001). Toll-like receptors: Critical proteins linking innate and acquired immunity. *Nat. Immunol.* 2 675–680. 10.1038/90609 11477402

[B3] Alagarasu K. Bachal R. Memane R. Shah P. Cecilia D. (2015). Polymorphisms in RNA sensing toll like receptor genes and its association with clinical outcomes of dengue virus infection. *Immunobiology* 220 164–168. 10.1016/j.imbio.2014.09.020 25446400

[B4] Alexopoulou L. Holt A. Medzhitov R. Flavell R. (2001). Recognition of double-stranded RNA and activation of NF-kappaB by toll-like receptor 3. *Nature* 413 732–738. 10.1038/35099560 11607032

[B5] Alhabibi A. Hassan A. Abd Elbaky N. Eid H. Khalifa M. Wahab M. (2023). Impact of toll-like receptor 2 and 9 gene polymorphisms on COVID-19: Susceptibility, severity, and thrombosis. *J. Inflamm. Res.* 16 665–675. 10.2147/JIR.S394927 36825132 PMC9942505

[B6] Alonso-Padilla J. de Oya N. Blázquez A. Escribano-Romero E. Escribano J. Saiz J. (2011). Recombinant West Nile virus envelope protein E and domain III expressed in insect larvae protects mice against West Nile disease. *Vaccine* 29 1830–1835. 10.1016/j.vaccine.2010.12.081 21211580

[B7] Anderson J. Andreadis T. Vossbrinck C. Tirrell S. Wakem E. French R. (1999). Isolation of West Nile virus from mosquitoes, crows, and a Cooper’s hawk in connecticut. *Science* 286 2331–2333. 10.1126/science.286.5448.2331 10600741

[B8] Andino R. Darling D. (2025). Now you see me, now you don’t: What and how viral RNAs are detected by cytoplasmic pattern-recognition receptors. *Mol. Cell.* 85 1482–1483. 10.1016/j.molcel.2025.03.019 40250409

[B9] Arjona A. Ledizet M. Anthony K. Bonafé N. Modis Y. Town T. (2007). West Nile virus envelope protein inhibits dsRNA-induced innate immune responses. *J. Immunol.* 179 8403–8409. 10.4049/jimmunol.179.12.8403 18056386

[B10] Behari J. Yadav K. Khare P. Kumar B. Kushwaha A. (2024). Recent insights on pattern recognition receptors and the interplay of innate immune responses against West Nile Virus infection. *Virology* 600:110267. 10.1016/j.virol.2024.110267 39437534

[B11] Biedenbender R. Bevilacqua J. Gregg A. Watson M. Dayan G. (2011). Phase II, randomized, double-blind, placebo-controlled, multicenter study to investigate the immunogenicity and safety of a West Nile virus vaccine in healthy adults. *J. Infect. Dis.* 203 75–84. 10.1093/infdis/jiq003 21148499 PMC3086439

[B12] Biyani S. Garg R. Jain A. Malhotra H. Kumar R. Prakash S. (2015). Toll-like receptor-3 gene polymorphism in patients with Japanese encephalitis. *J. Neuroimmunol.* 286 71–76. 10.1016/j.jneuroim.2015.07.010 26298326

[B13] Carter D. De La Rosa G. Garçon N. Moon H. Nam H. Skibinski D. (2025). The success of toll-like receptor 4 based vaccine adjuvants. *Vaccine* 61:127413. 10.1016/j.vaccine.2025.127413 40570747

[B14] Carty M. Bowie A. (2010). Recent insights into the role of Toll-like receptors in viral infection. *Clin. Exp. Immunol.* 161 397–406. 10.1111/j.1365-2249.2010.04196.x 20560984 PMC2962956

[B15] Carty M. Guy C. Bowie A. (2021). Detection of viral Infections by Innate Immunity. *Biochem. Pharmacol.* 183:114316. 10.1016/j.bcp.2020.114316 33152343

[B16] Chancey C. Grinev A. Volkova E. Rios M. (2015). The global ecology and epidemiology of West Nile virus. *Biomed. Res. Int.* 2015: 376230. 10.1155/2015/376230 25866777 PMC4383390

[B17] Chaturvedi A. Pierce S. (2009). How location governs toll-like receptor signaling. *Traffic* 10 621–628. 10.1111/j.1600-0854.2009.00899.x 19302269 PMC2741634

[B18] Choi K. Lee Y. Lim S. Choi H. Lee C. Lee E. (2006). Smad6 negatively regulates interleukin 1-receptor-Toll-like receptor signaling through direct interaction with the adaptor Pellino-1. *Nat. Immunol.* 7 1057–1065. 10.1038/ni1383 16951688

[B19] Chugh P. Damania B. Dittmer D. (2014). Toll-like receptor-3 is dispensable for the innate microRNA response to West Nile virus (WNV). *PLoS One* 9:e104770. 10.1371/journal.pone.0104770 25127040 PMC4134228

[B20] Chung K. Liszewski M. Nybakken G. Davis A. Townsend R. Fremont D. (2006). West Nile virus nonstructural protein NS1 inhibits complement activation by binding the regulatory protein factor H. *Proc. Natl. Acad. Sci. U S A.* 103 19111–19116. 10.1073/pnas.0605668103 17132743 PMC1664712

[B21] Colpitts T. Conway M. Montgomery R. Fikrig E. (2012). West Nile virus: Biology, transmission, and human infection. *Clin. Microbiol. Rev.* 25 635–648. 10.1128/CMR.00045-12 23034323 PMC3485754

[B22] Crawford M. Abdelwahab W. Siram K. Parkins C. Harrison H. Stone E. (2025). The TLR7/8 agonist INI-4001 enhances the immunogenicity of a Powassan virus-like-particle vaccine. *NPJ Vaccines* 10:156. 10.1038/s41541-025-01215-9 40670406 PMC12267508

[B23] Crook K. Miller-Kittrell M. Morrison C. Scholle F. (2014). Modulation of innate immune signaling by the secreted form of the West Nile virus NS1 glycoprotein. *Virology* 458-459 172–182. 10.1016/j.virol.2014.04.036 24928049 PMC4075170

[B24] Daep C. Muñoz-Jordán J. Eugenin E. (2014). Flaviviruses, an expanding threat in public health: Focus on dengue, West Nile, and Japanese encephalitis virus. *J. Neurovirol.* 20 539–560. 10.1007/s13365-014-0285-z 25287260 PMC4331079

[B25] Daffis S. Lazear H. Liu W. Audsley M. Engle M. Khromykh A. (2011). The naturally attenuated Kunjin strain of West Nile virus shows enhanced sensitivity to the host type I interferon response. *J. Virol.* 85 5664–5668. 10.1128/JVI.00232-11 21411525 PMC3094947

[B26] Daffis S. Samuel M. Keller B. Gale M. Diamond M. (2007). Cell-specific IRF-3 responses protect against West Nile virus infection by interferon-dependent and -independent mechanisms. *PLoS Pathog.* 3:e106. 10.1371/journal.ppat.0030106 17676997 PMC1933455

[B27] Daffis S. Samuel M. Suthar M. Gale M. Diamond M. (2008). Toll-like receptor 3 has a protective role against West Nile virus infection. *J. Virol.* 82 10349–10358. 10.1128/JVI.00935-08 18715906 PMC2573187

[B28] Davis E. Velez J. Hamik J. Fitzpatrick K. Haley J. Eschliman J. (2024). Evidence of lineage 1 and 3 West Nile Virus in person with neuroinvasive disease, Nebraska, USA, 2023. *Emerg. Infect. Dis.* 30 2090–2098. 10.3201/eid3010.240595 39320165 PMC11431902

[B29] De Filette M. Chabierski S. Andries O. Ulbert S. Sanders N. N. (2014a). T cell epitope mapping of the e-protein of West Nile virus in BALB/c mice. *PLoS One* 9:e115343. 10.1371/journal.pone.0115343 25506689 PMC4266646

[B30] De Filette M. Soehle S. Ulbert S. Richner J. Diamond M. Sinigaglia A. (2014b). Vaccination of mice using the West Nile virus E-protein in a DNA prime-protein boost strategy stimulates cell-mediated immunity and protects mice against a lethal challenge. *PLoS One* 9:e87837. 10.1371/journal.pone.0087837 24503579 PMC3913677

[B31] Deguine J. Barton G. (2014). MyD88: A central player in innate immune signaling. *F1000Prime Rep.* 6:97. 10.12703/P6-97 25580251 PMC4229726

[B32] Demento S. Bonafé N. Cui W. Kaech S. Caplan M. Fikrig E. (2010). TLR9-targeted biodegradable nanoparticles as immunization vectors protect against West Nile encephalitis. *J. Immunol.* 185 2989–2997. 10.4049/jimmunol.1000768 20660705 PMC3753007

[B33] Diamond M. Kanneganti T. (2022). Innate immunity: The first line of defense against SARS-CoV-2. *Nat. Immunol.* 23 165–176. 10.1038/s41590-021-01091-0 35105981 PMC8935980

[B34] Diamond M. Mehlhop E. Oliphant T. Samuel M. (2009). The host immunologic response to West Nile encephalitis virus. *Front. Biosci.* 14:3024–3034. 10.2741/3432 19273254

[B35] Diebold S. Kaisho T. Hemmi H. Akira S. Reis e Sousa C. (2004). Innate antiviral responses by means of TLR7-mediated recognition of single-stranded RNA. *Science* 303 1529–1531. 10.1126/science.1093616 14976261

[B36] Du Y. Deng Y. Zhan Y. Yang R. Ren J. Wang W. (2023a). The recombinant truncated envelope protein of West Nile virus adjuvanted with Alum/CpG induces potent humoral and T cell immunity in mice. *Biosaf. Health* 5 300–307. 10.1016/j.bsheal.2023.06.003 40078908 PMC11894981

[B37] Du Y. Li S. Wang X. Liu J. Gao Y. Lv W. (2023b). Corrigendum: Meta-analysis of the association between toll-like receptor gene polymorphisms and hepatitis C virus infection. *Front. Microbiol.* 14:1330170. 10.3389/fmicb.2023.1330170 38075931 PMC10698469

[B38] Durbin A. Wright P. Cox A. Kagucia W. Elwood D. Henderson S. (2013). The live attenuated chimeric vaccine rWN/DEN4Δ30 is well-tolerated and immunogenic in healthy flavivirus-naïve adult volunteers. *Vaccine* 31 5772–5777. 10.1016/j.vaccine.2013.07.064 23968769 PMC3833717

[B39] El Garch H. Minke J. Rehder J. Richard S. Edlund Toulemonde C. Dinic S. (2008). A West Nile virus (WNV) recombinant canarypox virus vaccine elicits WNV-specific neutralizing antibodies and cell-mediated immune responses in the horse. *Vet. Immunol. Immunopathol.* 123 230–239. 10.1016/j.vetimm.2008.02.002 18372050

[B40] Errett J. Suthar M. McMillan A. Diamond M. Gale M. (2013). The essential, nonredundant roles of RIG-I and MDA5 in detecting and controlling West Nile virus infection. *J. Virol.* 87 11416–11425. 10.1128/JVI.01488-13 23966395 PMC3807316

[B41] Fitzgerald K. Kagan J. (2020). Toll-like receptors and the control of immunity. *Cell* 180 1044–1066. 10.1016/j.cell.2020.02.041 32164908 PMC9358771

[B42] Formosinho P. Santos-Silva M. (2006). Experimental infection of Hyalomma marginatum ticks with West Nile virus. *Acta Virol.* 50 175–180. 17131936

[B43] Fredericksen B. Gale M. (2006). West Nile virus evades activation of interferon regulatory factor 3 through RIG-I-dependent and -independent pathways without antagonizing host defense signaling. *J. Virol.* 80 2913–2923. 10.1128/JVI.80.6.2913-2923.2006 16501100 PMC1395472

[B44] Fulton C. Beasley D. Bente D. Dineley K. (2020). Long-term, West Nile virus-induced neurological changes: A comparison of patients and rodent models. *Brain Behav. Immun. Health* 7:100105. 10.1016/j.bbih.2020.100105 34589866 PMC8474605

[B45] Genoyer E. Wilson J. Ames J. Stokes C. Moreno D. Etzyon N. (2025). Exposure of negative-sense viral RNA in the cytoplasm initiates innate immunity to West Nile virus. *Mol. Cell.* 85 1147–1161.e9. 10.1016/j.molcel.2025.01.015 39919747 PMC11931551

[B46] Gould C. Staples J. Huang C. Brault A. Nett R. (2023). Combating West Nile virus disease - time to revisit vaccination. *N. Engl. J. Med.* 388 1633–1636. 10.1056/NEJMp2301816 37125778 PMC11627013

[B47] Goulet M. Olagnier D. Xu Z. Paz S. Belgnaoui S. Lafferty E. (2013). Systems analysis of a RIG-I agonist inducing broad spectrum inhibition of virus infectivity. *PLoS Pathog.* 9:e1003298. 10.1371/journal.ppat.1003298 23633948 PMC3635991

[B48] Green R. Wilkins C. Thomas S. Sekine A. Hendrick D. Voss K. (2017). Oas1b-dependent immune transcriptional profiles of West Nile Virus infection in the collaborative cross. *G3* 7 1665–1682. 10.1534/g3.117.041624 28592649 PMC5473748

[B49] Hajam I. Dar P. Shahnawaz I. Jaume J. Lee J. (2017). Bacterial flagellin-a potent immunomodulatory agent. *Exp. Mol. Med.* 49:e373. 10.1038/emm.2017.172 28860663 PMC5628280

[B50] He J. Peng L. Lai H. Hurtado J. Stahnke J. Chen Q. (2014). A plant-produced antigen elicits potent immune responses against West Nile virus in mice. *Biomed. Res. Int.* 2014:952865. 10.1155/2014/952865 24804264 PMC3996298

[B51] Heim M. Thimme R. (2014). Innate and adaptive immune responses in HCV infections. *J. Hepatol.* 61 S14–S25. 10.1016/j.jhep.2014.06.035 25443342

[B52] Huang B. Zhao J. Unkeless J. Feng Z. Xiong H. (2008). TLR signaling by tumor and immune cells: A double-edged sword. *Oncogene* 27 218–224. 10.1038/sj.onc.1210904 18176603

[B53] Hutcheson H. Gorham C. Machain-Williams C. Loroño-Pino M. James A. Marlenee N. (2005). Experimental transmission of West Nile virus (Flaviviridae: flavivirus) by Carios capensis ticks from North America. *Vector Borne Zoonotic Dis.* 5 293–295. 10.1089/vbz.2005.5.293 16187900

[B54] Iwasaki A. Omer S. (2020). Why and how vaccines work. *Cell* 183 290–295. 10.1016/j.cell.2020.09.040 33064982 PMC7560117

[B55] Iyer A. Pahar B. Boudreaux M. Wakamatsu N. Roy A. Chouljenko V. (2009). Recombinant vesicular stomatitis virus-based west Nile vaccine elicits strong humoral and cellular immune responses and protects mice against lethal challenge with the virulent west Nile virus strain LSU-AR01. *Vaccine* 27 893–903. 10.1016/j.vaccine.2008.11.087 19070640 PMC7115407

[B56] Kaiser J. Barrett A. (2019). Twenty years of progress toward West Nile Virus vaccine development. *Viruses* 11:823. 10.3390/v11090823 31491885 PMC6784102

[B57] Kawai T. Akira S. (2008). Toll-like receptor and RIG-I-like receptor signaling. *Ann. N. Y. Acad. Sci.* 1143 1–20. 10.1196/annals.1443.020 19076341

[B58] Kawai T. Ikegawa M. Ori D. Akira S. (2024). Decoding toll-like receptors: Recent insights and perspectives in innate immunity. *Immunity* 57 649–673. 10.1016/j.immuni.2024.03.004 38599164

[B59] Kawasaki T. Kawai T. (2014). Toll-like receptor signaling pathways. *Front. Immunol.* 5:461. 10.3389/fimmu.2014.00461 25309543 PMC4174766

[B60] Kayesh M. Kohara M. Tsukiyama-Kohara K. (2021). An overview of recent insights into the response of TLR to SARS-CoV-2 infection and the potential of TLR agonists as SARS-CoV-2 vaccine adjuvants. *Viruses* 13:2302. 10.3390/v13112302 34835108 PMC8622245

[B61] Kayesh M. Kohara M. Tsukiyama-Kohara K. (2023). TLR agonists as vaccine adjuvants in the prevention of viral infections: An overview. *Front. Microbiol.* 14:1249718. 10.3389/fmicb.2023.1249718 38179453 PMC10764465

[B62] Kayesh M. Kohara M. Tsukiyama-Kohara K. (2025). Innate immune response to powassan virus infection: Progress toward infection control. *Vaccines* 13:754. 10.3390/vaccines13070754 40733731 PMC12299467

[B63] Keller B. Fredericksen B. Samuel M. Mock R. Mason P. Diamond M. (2006). Resistance to alpha/beta interferon is a determinant of West Nile virus replication fitness and virulence. *J. Virol.* 80 9424–9434. 10.1128/JVI.00768-06 16973548 PMC1617238

[B64] Kocabiyik D. Álvarez L. Durigon E. Wrenger C. (2025). West Nile virus - a re-emerging global threat: Recent advances in vaccines and drug discovery. *Front. Cell. Infect. Microbiol.* 15:1568031. 10.3389/fcimb.2025.1568031 40444156 PMC12119551

[B65] Koch R. Erazo D. Folly A. Johnson N. Dellicour S. Grubaugh N. (2024). Genomic epidemiology of West Nile virus in Europe. *One Health* 18:100664. 10.1016/j.onehlt.2023.100664 38193029 PMC10772404

[B66] Kong K. Delroux K. Wang X. Qian F. Arjona A. Malawista S. (2008). Dysregulation of TLR3 impairs the innate immune response to West Nile virus in the elderly. *J. Virol.* 82 7613–7623. 10.1128/JVI.00618-08 18508883 PMC2493309

[B67] Kramer L. Li J. Shi P. (2007). West Nile virus. *Lancet Neurol.* 6 171–181. 10.1016/S1474-4422(07)70030-3 17239804

[B68] Kumar H. Kawai T. Kato H. Sato S. Takahashi K. Coban C. (2006). Essential role of IPS-1 in innate immune responses against RNA viruses. *J. Exp. Med.* 203 1795–1803. 10.1084/jem.20060792 16785313 PMC2118350

[B69] Lai H. Engle M. Fuchs A. Keller T. Johnson S. Gorlatov S. (2010). Monoclonal antibody produced in plants efficiently treats West Nile virus infection in mice. *Proc. Natl. Acad. Sci. U S A.* 107 2419–2424. 10.1073/pnas.0914503107 20133644 PMC2823901

[B70] Lanciotti R. Roehrig J. Deubel V. Smith J. Parker M. Steele K. (1999). Origin of the West Nile virus responsible for an outbreak of encephalitis in the northeastern United States. *Science* 286 2333–2337. 10.1126/science.286.5448.2333 10600742

[B71] Laurent-Rolle M. Boer E. Lubick K. Wolfinbarger J. Carmody A. Rockx B. (2010). The NS5 protein of the virulent West Nile virus NY99 strain is a potent antagonist of type I interferon-mediated JAK-STAT signaling. *J. Virol.* 84 3503–3515. 10.1128/JVI.01161-09 20106931 PMC2838099

[B72] Lazear H. Pinto A. Ramos H. Vick S. Shrestha B. Suthar M. (2013). Pattern recognition receptor MDA5 modulates CD8+ T cell-dependent clearance of West Nile virus from the central nervous system. *J. Virol.* 87 11401–11415. 10.1128/JVI.01403-13 23966390 PMC3807324

[B73] Lazear H. Pinto A. Vogt M. Gale M. Diamond M. (2011). Beta interferon controls West Nile virus infection and pathogenesis in mice. *J. Virol.* 85 7186–7194. 10.1128/JVI.00396-11 21543483 PMC3126609

[B74] Ledgerwood J. Pierson T. Hubka S. Desai N. Rucker S. Gordon I. (2011). A West Nile virus DNA vaccine utilizing a modified promoter induces neutralizing antibody in younger and older healthy adults in a phase I clinical trial. *J. Infect. Dis.* 203 1396–1404. 10.1093/infdis/jir054 21398392 PMC3080891

[B75] Lee M. Kim Y. (2007). Signaling pathways downstream of pattern-recognition receptors and their cross talk. *Annu. Rev. Biochem.* 76 447–480. 10.1146/annurev.biochem.76.060605.122847 17328678

[B76] Lee S. Kok K. Jaume M. Cheung T. Yip T. Lai J. (2014). Toll-like receptor 10 is involved in induction of innate immune responses to influenza virus infection. *Proc. Natl. Acad. Sci. U S A.* 111 3793–3798. 10.1073/pnas.1324266111 24567377 PMC3956146

[B77] Lester S. Li K. (2014). Toll-like receptors in antiviral innate immunity. *J. Mol. Biol.* 426 1246–1264. 10.1016/j.jmb.2013.11.024 24316048 PMC3943763

[B78] Lim S. Koraka P. Osterhaus A. Martina B. (2011). West Nile virus: Immunity and pathogenesis. *Viruses* 3 811–828. 10.3390/v3060811 21994755 PMC3185772

[B79] Liu W. Wang X. Mokhonov V. Shi P. Randall R. Khromykh A. (2005). Inhibition of interferon signaling by the New York 99 strain and Kunjin subtype of West Nile virus involves blockage of STAT1 and STAT2 activation by nonstructural proteins. *J. Virol.* 79 1934–1942. 10.1128/JVI.79.3.1934-1942.2005 15650219 PMC544092

[B80] Luo H. Li G. Wang B. Tian B. Gao J. Zou J. (2020). Peli1 signaling blockade attenuates congenital zika syndrome. *PLoS Pathog.* 16:e1008538. 10.1371/journal.ppat.1008538 32544190 PMC7297310

[B81] Luo H. Winkelmann E. Zhu S. Ru W. Mays E. Silvas J. (2018). Peli1 facilitates virus replication and promotes neuroinflammation during West Nile virus infection. *J. Clin. Invest.* 128 4980–4991. 10.1172/JCI99902 30247157 PMC6205407

[B82] Marshall J. Warrington R. Watson W. Kim H. (2018). An introduction to immunology and immunopathology. *Allergy Asthma Clin. Immunol.* 14:49. 10.1186/s13223-018-0278-1 30263032 PMC6156898

[B83] Martin J. Pierson T. Hubka S. Rucker S. Gordon I. Enama M. (2007). A West Nile virus DNA vaccine induces neutralizing antibody in healthy adults during a phase 1 clinical trial. *J. Infect. Dis.* 196 1732–1740. 10.1086/523650 18190252 PMC2714735

[B84] McDonald W. Huleatt J. Foellmer H. Hewitt D. Tang J. Desai P. (2007). A West Nile virus recombinant protein vaccine that coactivates innate and adaptive immunity. *J. Infect. Dis.* 195 1607–1617. 10.1086/517613 17471430

[B85] Medvedev A. (2013). Toll-like receptor polymorphisms, inflammatory and infectious diseases, allergies, and cancer. *J. Interferon Cytokine Res.* 33 467–484. 10.1089/jir.2012.0140 23675778 PMC3760066

[B86] Medzhitov R. Janeway C. (2000). Innate immunity. *N. Engl. J. Med.* 343 338–344. 10.1056/NEJM200008033430506 10922424

[B87] Modhiran N. Watterson D. Muller D. Panetta A. Sester D. Liu L. (2015). Dengue virus NS1 protein activates cells via Toll-like receptor 4 and disrupts endothelial cell monolayer integrity. *Sci. Transl. Med.* 7:304ra142. 10.1126/scitranslmed.aaa3863 26355031

[B88] Mogensen T. (2009). Pathogen recognition and inflammatory signaling in innate immune defenses. *Clin. Microbiol. Rev.* 22 240–273. 10.1128/CMR.00046-08 19366914 PMC2668232

[B89] Monath T. Liu J. Kanesa-Thasan N. Myers G. Nichols R. Deary A. (2006). A live, attenuated recombinant West Nile virus vaccine. *Proc. Natl. Acad. Sci. U S A.* 103 6694–6699. 10.1073/pnas.0601932103 16617103 PMC1436023

[B90] Oh D. Baumann K. Hamouda O. Eckert J. Neumann K. Kücherer C. (2009). A frequent functional toll-like receptor 7 polymorphism is associated with accelerated HIV-1 disease progression. *AIDS* 23 297–307. 10.1097/QAD.0b013e32831fb540 19114863

[B91] Okuzumi S. Miyata J. Kabata H. Mochimaru T. Kagawa S. Masaki K. (2021). TLR7 agonist suppresses group 2 innate lymphoid cell-mediated inflammation via IL-27-producing interstitial macrophages. *Am J Respir Cell. Mol. Biol.* 65 309–318. 10.1165/rcmb.2021-0042OC 34003734

[B92] Parsania M. Khorrami S. Hasanzad M. Parsania N. Nagozir S. Mokhtari N. (2024). Association of polymorphisms in TLR3 and TLR7 genes with susceptibility to COVID-19 among Iranian population: A retrospective case-control study. *Iran J. Microbiol.* 16 114–123. 10.18502/ijm.v16i1.14880 38682063 PMC11055434

[B93] Patel S. Sinigaglia A. Barzon L. Fassan M. Sparber F. LeibundGut-Landmann S. (2019). Role of NS1 and TLR3 in pathogenesis and immunity of WNV. *Viruses* 11:603. 10.3390/v11070603 31277274 PMC6669597

[B94] Paul A. Acharya D. Le L. Stokic D. Leis A. Bai F. (2015). Toll-like receptor 8 signaling facilitates West Nile virus infection in mice (VIR9P.1161). *J Immunol.* 194 215–227. 10.4049/jimmunol.194.Supp.215.27

[B95] Pierce K. Whitehead S. Kirkpatrick B. Grier P. Jarvis A. Kenney H. (2017). A live attenuated chimeric West Nile Virus vaccine, rWN/DEN4Δ30, is well tolerated and immunogenic in flavivirus-Naive older adult volunteers. *J. Infect. Dis.* 215 52–55. 10.1093/infdis/jiw501 28077583 PMC5225253

[B96] Pletnev A. Putnak R. Speicher J. Wagar E. Vaughn D. (2002). West Nile virus/dengue type 4 virus chimeras that are reduced in neurovirulence and peripheral virulence without loss of immunogenicity or protective efficacy. *Proc. Natl. Acad. Sci. U S A.* 99 3036–3041. 10.1073/pnas.022652799 11880643 PMC122468

[B97] Reed S. Orr M. Fox C. (2013). Key roles of adjuvants in modern vaccines. *Nat. Med.* 19 1597–1608. 10.1038/nm.3409 24309663

[B98] Sabouri A. Marcondes M. Flynn C. Berger M. Xiao N. Fox H. (2014). TLR signaling controls lethal encephalitis in WNV-infected brain. *Brain Res.* 1574 84–95. 10.1016/j.brainres.2014.05.049 24928618 PMC4099315

[B99] Saiz J. (2020). Animal and human vaccines against West Nile Virus. *Pathogens* 9:1073. 10.3390/pathogens9121073 33371384 PMC7767344

[B100] Samuel M. Diamond M. (2005). Alpha/beta interferon protects against lethal West Nile virus infection by restricting cellular tropism and enhancing neuronal survival. *J. Virol.* 79 13350–13361. 10.1128/JVI.79.21.13350-13361.2005 16227257 PMC1262587

[B101] Santos C. Magalhães L. Fonseca A. Bispo A. Porto R. Alves J. (2023). Association between genetic variants in TREM1, CXCL10, IL4, CXCL8 and TLR7 genes with the occurrence of congenital Zika syndrome and severe microcephaly. *Sci. Rep.* 13:3466. 10.1038/s41598-023-30342-3 36859461 PMC9975867

[B102] Schneeweiss A. Chabierski S. Salomo M. Delaroque N. Al-Robaiy S. Grunwald T. (2011). A DNA vaccine encoding the E protein of West Nile virus is protective and can be boosted by recombinant domain DIII. *Vaccine* 29 6352–6357. 10.1016/j.vaccine.2011.04.116 21596075

[B103] Schoggins J. MacDuff D. Imanaka N. Gainey M. Shrestha B. Eitson J. (2015). Corrigendum: Pan-viral specificity of IFN-induced genes reveals new roles for cGAS in innate immunity. *Nature* 525:144. 10.1038/nature14555 26153856 PMC8323779

[B104] Schoggins J. Wilson S. Panis M. Murphy M. Jones C. Bieniasz P. (2011). A diverse range of gene products are effectors of the type I interferon antiviral response. *Nature* 472 481–485. 10.1038/nature09907 21478870 PMC3409588

[B105] Scholle F. Mason P. (2005). West Nile virus replication interferes with both poly(I:c)-induced interferon gene transcription and response to interferon treatment. *Virology* 342 77–87. 10.1016/j.virol.2005.07.021 16111732

[B106] Sejvar J. (2014). Clinical manifestations and outcomes of West Nile virus infection. *Viruses* 6 606–623. 10.3390/v6020606 24509812 PMC3939474

[B107] Siirin M. Newman P. Weeks-Levy C. Coller B. A. Xiao S. Y. (2008). Travassos da Rosa AP. *Am. J. Trop. Med. Hyg.* 79 955–962. 19052311 PMC2765405

[B108] Smithburn K. Hughes T. Burke A. (1940). A neurotropic virus isolated from the blood of a native of Uganda. *Am. J. Trop. Med. Hygiene* s1-20 471–492. 10.4269/ajtmh.1940.s1-20.471

[B109] Spohn G. Jennings G. Martina B. Keller I. Beck M. Pumpens P. (2010). A VLP-based vaccine targeting domain III of the West Nile virus E protein protects from lethal infection in mice. *Virol. J.* 7:146. 10.1186/1743-422X-7-146 20604940 PMC2914671

[B110] Sultana H. Neelakanta G. Foellmer H. Montgomery R. Anderson J. Koski R. (2012). Semaphorin 7A contributes to West Nile virus pathogenesis through TGF-β1/Smad6 signaling. *J. Immunol.* 189 3150–3158. 10.4049/jimmunol.1201140 22896629 PMC3496209

[B111] Sun H. Acharya D. Paul A. Lai H. He J. Bai F. (2023). Antibody-dependent enhancement activity of a plant-made vaccine against West Nile Virus. *Vaccines* 11:197. 10.3390/vaccines11020197 36851075 PMC9966755

[B112] Suthar M. Ma D. Thomas S. Lund J. Zhang N. Daffis S. (2010). IPS-1 is essential for the control of West Nile virus infection and immunity. *PLoS Pathog.* 6:e1000757. 10.1371/journal.ppat.1000757 20140199 PMC2816698

[B113] Szretter K. Daffis S. Patel J. Suthar M. Klein R. Gale M. (2010). The innate immune adaptor molecule MyD88 restricts West Nile virus replication and spread in neurons of the central nervous system. *J. Virol.* 84 12125–12138. 10.1128/JVI.01026-10 20881045 PMC2976388

[B114] Town T. Bai F. Wang T. Kaplan A. Qian F. Montgomery R. (2009). Toll-like receptor 7 mitigates lethal West Nile encephalitis via interleukin 23-dependent immune cell infiltration and homing. *Immunity* 30 242–253. 10.1016/j.immuni.2008.11.012 19200759 PMC2707901

[B115] Van Hoeven N. Joshi S. Nana G. Bosco-Lauth A. Fox C. Bowen R. (2016). A Novel Synthetic TLR-4 agonist adjuvant increases the protective response to a clinical-stage West Nile Virus vaccine antigen in multiple formulations. *PLoS One* 11:e0149610. 10.1371/journal.pone.0149610 26901122 PMC4762984

[B116] Van Hoeven N. Wiley S. Gage E. Fiore-Gartland A. Granger B. Gray S. (2018). A combination of TLR-4 agonist and saponin adjuvants increases antibody diversity and protective efficacy of a recombinant West Nile Virus antigen. *NPJ Vaccines* 3:39. 10.1038/s41541-018-0077-1 30302281 PMC6158298

[B117] Vorovitch M. Tuchynskaya K. Kruglov Y. Peunkov N. Mostipanova G. Kholodilov I. (2024). An inactivated West Nile Virus vaccine candidate based on the Lineage 2 strain. *Vaccines* 12:1398. 10.3390/vaccines12121398 39772058 PMC11680355

[B118] Wang L. Yang L. Fikrig E. Wang P. (2017). An essential role of PI3K in the control of West Nile virus infection. *Sci. Rep.* 7:3724. 10.1038/s41598-017-03912-5 28623344 PMC5473900

[B119] Wang T. Town T. Alexopoulou L. Anderson J. Fikrig E. Flavell R. (2004). Toll-like receptor 3 mediates West Nile virus entry into the brain causing lethal encephalitis. *Nat. Med.* 10 1366–1373. 10.1038/nm1140 15558055

[B120] Weiß R. Issmail L. Rockstroh A. Grunwald T. Fertey J. Ulbert S. (2023). Immunization with different recombinant West Nile virus envelope proteins induces varying levels of serological cross-reactivity and protection from infection. *Front. Cell. Infect. Microbiol.* 13:1279147. 10.3389/fcimb.2023.1279147 38035335 PMC10684968

[B121] Welte T. Reagan K. Fang H. Machain-Williams C. Zheng X. Mendell N. (2009). Toll-like receptor 7-induced immune response to cutaneous West Nile virus infection. *J. Gen. Virol.* 90 2660–2668. 10.1099/vir.0.011783-0 19641044 PMC2771433

[B122] Wilson J. de Sessions P. Leon M. Scholle F. (2008). West Nile virus nonstructural protein 1 inhibits TLR3 signal transduction. *J .Virol.* 82 8262–8271. 10.1128/JVI.00226-08 18562533 PMC2519649

[B123] Woods C. Sanchez A. Swamy G. McClain M. Harrington L. Freeman D. (2019). An observer blinded, randomized, placebo-controlled, phase I dose escalation trial to evaluate the safety and immunogenicity of an inactivated West Nile virus *Vaccine*, HydroVax-001, in healthy adults. *Vaccine* 37 4222–4230. 10.1016/j.vaccine.2018.12.026 30661836 PMC6640644

[B124] Xia J. Winkelmann E. Gorder S. Mason P. Milligan G. (2013). TLR3- and MyD88-dependent signaling differentially influences the development of West Nile virus-specific B cell responses in mice following immunization with RepliVAX WN, a single-cycle flavivirus vaccine candidate. *J. Virol.* 87 12090–12101. 10.1128/JVI.01469-13 23986602 PMC3807881

[B125] Xie G. Luo H. Pang L. Peng B. Winkelmann E. McGruder B. (2016). Dysregulation of toll-like receptor 7 compromises innate and adaptive T cell responses and host resistance to an attenuated West Nile virus infection in old mice. *J. Virol.* 90 1333–1344. 10.1128/JVI.02488-15 26581984 PMC4719598

[B126] Xie G. Luo H. Tian B. Mann B. Bao X. McBride J. (2015). A West Nile virus NS4B-P38G mutant strain induces cell intrinsic innate cytokine responses in human monocytic and macrophage cells. *Vaccine* 33 869–878. 10.1016/j.vaccine.2014.12.056 25562791 PMC4312757

[B127] Xie G. Welte T. Wang J. Whiteman M. Wicker J. Saxena V. (2013). A West Nile virus NS4B-P38G mutant strain induces adaptive immunity via TLR7-MyD88-dependent and independent signaling pathways. *Vaccine* 31 4143–4151. 10.1016/j.vaccine.2013.06.093 23845800 PMC3870894

[B128] Yokota S. Okabayashi T. Fujii N. (2010). The battle between virus and host: Modulation of Toll-like receptor signaling pathways by virus infection. *Mediators Inflamm.* 2010:184328. 10.1155/2010/184328 20672047 PMC2903949

[B129] You F. Wang P. Yang L. Yang G. Zhao Y. Qian F. (2013). ELF4 is critical for induction of type I interferon and the host antiviral response. *Nat. Immunol.* 14 1237–1246. 10.1038/ni.2756 24185615 PMC3939855

[B130] Zeller H. Schuffenecker I. (2004). West Nile virus: An overview of its spread in Europe and the Mediterranean basin in contrast to its spread in the Americas. *Eur. J. Clin. Microbiol. Infect. Dis.* 23 147–156. 10.1007/s10096-003-1085-1 14986160

[B131] Zhang H. Ye H. Liu S. Deng C. Li X. Shi P. (2017). West Nile Virus NS1 antagonizes interferon beta production by targeting RIG-I and MDA5. *J. Virol.* 91:e02396-16. 10.1128/JVI.02396-16 28659477 PMC5571242

[B132] Zhang S. Jouanguy E. Ugolini S. Smahi A. Elain G. Romero P. (2007). TLR3 deficiency in patients with herpes simplex encephalitis. *Science* 317 1522–1527. 10.1126/science.1139522 17872438

[B133] Zhao T. Cai Y. Jiang Y. He X. Wei Y. Yu Y. (2023). Vaccine adjuvants: Mechanisms and platforms. *Signal Transduct Target Ther.* 8:283. 10.1038/s41392-023-01557-7 37468460 PMC10356842

[B134] Zimmerman M. Bowen J. McDonald C. Pulendran B. Suthar M. (2019). West Nile virus infection blocks inflammatory response and T cell costimulatory capacity of human monocyte-derived dendritic cells. *J. Virol.* 93:e00664-19. 10.1128/JVI.00664-19 31534040 PMC6854506

[B135] Zipfel P. Skerka C. Hellwage J. Jokiranta S. Meri S. Brade V. (2002). Factor H family proteins: On complement, microbes and human diseases. *Biochem. Soc. Trans.* 30 971–978. 10.1042/bst0300971 12440956

